# Primary Hyperammonaemia: Current Diagnostic and Therapeutic Strategies

**DOI:** 10.34763/jmotherandchild.20202402si.2015.000006

**Published:** 2020-10-02

**Authors:** Johannes Häberle

**Affiliations:** 1 Division of Metabolism and Children’s Research Center, University Children’s Hospital Zurich, Zurich, Switzerland

**Keywords:** hyperammonaemia, urea cycle, nitrogen metabolism, encephalopathy, haemodialysis, low-protein diet, nitrogen scavengers

## Abstract

Primary hyperammonaemia is a term to describe an elevation of ammonia in blood or plasma due to a defect within the urea cycle, which is the pathway responsible for ammonia detoxification and arginine biosynthesis. Urea cycle disorders (UCDs) are rare diseases caused by genetic defects affecting any of the six enzymes or two transporters that are directly involved in the urea cycle function.

The clinical situation is variable and largely depends on the time of onset. Newborns who are often affected by hyper-ammonaemic encephalopathy carry a potential risk of severe brain damage, which may lead to death. Outside the neonatal period, symptoms are very unspecific but most often neurological (with wide variability), psychiatric and/or gastrointestinal. Early identification of patients is extremely important to start effective treatment modalities immediately. The acute management includes detoxification of ammonia, which often requires extracorporeal means such as haemodialysis, and the use of intravenous drugs that work as nitrogen scavengers. Long-term management of patients with UCDs consists of a low-protein diet, which needs to be balanced and supplemented to avoid deficiencies of essential amino acids, trace elements or vitamins and the use of nitrogen scavengers.

The reader will find here a brief overview describing the most relevant aspects of the clinical management of UCDs in an attempt to raise awareness for this important group of rare diseases.

## Introduction

The urea cycle is a metabolic pathway responsible for the detoxification of surplus nitrogenous metabolites and the endogenous synthesis of arginine (1). If this cycle is impaired in function or entirely defective, then neurotoxic ammonia will increase in all body fluids, and hence, there is a risk for arginine deficiency. The complete cycle is localised exclusively in periportal liver cells, a compartment where portal blood with high concentrations of ammonia from enteral protein degradation arrives at the liver (2). The proximal part of the urea cycle besides is expressed in the small intestine and contributes to citrulline biosynthesis in this organ but not the removal of ammonia.

**Figure 1:**
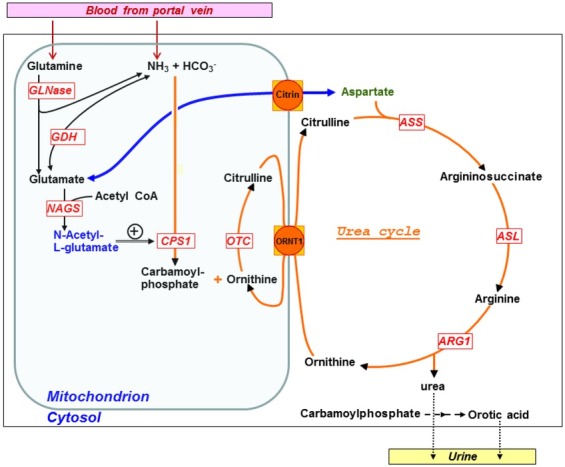
Simplified graph showing the urea cycle in mitochondrion and cytosol of hepatocytes. ARG1, arginase 1; ASL, argininosuccinate lyase; ASS, argininosuccinate synthetase; CPS1, carbamoylphosphate synthetase 1; GDH, glutamate dehydrogenase; GLNase, glutaminase; NAGS, N-acetylglutamate synthase; ORNT1, mitochondrial ornithine transporter (ornithine/citrulline antiporter); OTC, ornithine transcarbamylase.

The distribution of the six enzymes, which comprise the urea cycle in mitochondria and cytosol and the requirement of substrate supply across different cell compartments, renders necessary the presence of transporters (Figure 1). They function as antiporters and exchange aspartate for glutamate (Citrin) and citrulline for ornithine (ORNT1).

Urea cycle disorders (UCDs) are rare genetic diseases and follow an autosomal-recessive trait in all cases besides the most frequent ornithine transcarbamylase (OTC) deficiency that is X-linked (3). Thus, the presence of consanguinity in the family increases the risk for (not only) this group of metabolic disorders, and hence a careful study of the family history is prudent (4). Parents of the affected patients are obligate carriers of the disease-causing variants but do not show clinical or biochemical signs or symptoms. In the case of OTC deficiency, the situation is more complex and dependents on variable X-inactivation. There is a wide clinical spectrum in heterozygous females ranging from asymptomatic to severely affected, while males usually present with the severe disease early in life (5, 6).

Treatment is possible but no cure (4). The first aim in all symptomatic patients is to conduct rapid ammonia detoxification to prevent further brain damage. This can be achieved by several medications but often requires the additional use of extracorporeal means including haemodiafiltration or haemodialysis. Long-term management consists of a low-protein diet supplement with essential amino acids, trace elements and vitamins and the use of nitrogen scavenging drugs. In severe cases, where there are risks for recurrent hyperammonaemic encephalopathy, liver transplantation is recommended before severe brain damage occurs.

The prognosis largely depends on the severity and the duration of the initial (and possibly subsequent) hyperammonaemic decompensation(s). This highlights the importance of early identification of hyperammonaemia in patients, and an immediate start of effective treatment measures. This article aims to increase the awareness to the reader about this rare but often fatal group of metabolic disorders.

## Definition

Primary hyperammonaemia is a term that describes an elevation of ammonia in blood or plasma due to a defect within the urea cycle, that is, any of the enzymes or transporters directly involved in this pathway. In contrast, secondary hyperammonaemia can be caused by inherited or acquired conditions that indirectly affect the urea cycle (7). The most common aetiologies of secondary hyperammonaemia are inhibition of enzymes involved in the urea cycle or substrate deficiencies leading to functional urea cycle impairment.

The urea cycle comprises at least six enzymes and two transporters (shown in Figure 1) (1). There are mutations known in all the respective genes, which result in complete or partial loss of enzyme or transporter function and therefore the impairment of ureagenesis. UCDs are rare diseases with a cumulative incidence of 1 in 35,000 (8). However, some UCDs are far less prevalent, with N-acetylglutamate synthase (NAGS) deficiency being the rarest form (9). In some populations, for instance in Japan and other countries in the Far East, citrin deficiency exhibits a relatively high incidence of up to 1 in 17,000 (10, 11). The most common UCD globally is the OTC deficiency related to X-linked conditions, while all other UCDs are autosomal recessive diseases (12). This condition affects both sexes and females can present as severe as males, even with the early-onset disease in newborns.

## Pathophysiology

The common biochemical trait of all UCDs is hyperammonaemia that causes brain damage. Ammonia is a potent neurotoxin that can freely diffuse across the blood–brain barrier with severe consequences on normal brain function, including mainly brain oedema due to the accumulation of glutamine (13). This usually has the highest concentration of all amino acids in our blood and acts as an osmolyte leading to astrocytic water retention, changes of glutamate and N-methyl-d-aspartate (NMDA)-mediated neurotransmission and brain mitochondrial function. The reader is referred here to some recent reviews that deal with ammonia neurotoxicity in detail (14, 15).

All therapies of primary hyperammonaemia aim to either prevent the occurrence of hyperammonaemia or lead to a rapid reduction and detoxification of ammonia.

### Clinical Aspects

All UCDs can manifest at all ages. Nevertheless, about half of the patients present early in life during the neonatal period (16, 17). These patients are classified as ‘early-onset UCDs’. Another about 50% of the patients present outside the neonatal period, that is, later than the first 28 days in life and are called ‘late-onset UCD patients’. It is generally accepted that a complete loss of enzyme or transporter function very likely leads to the neonatal onset and severe disease. However, there is also significant morbidity and mortality in late-onset patients (18, 19, 20). In particular, there is a relevant risk of death during the first presentation both for neonatal and late-onset forms. Early identification of the affected patients and the immediate start of therapy are of utmost importance to treat UCDs.

The symptoms in both forms are mainly neurological and in most patients not very specific (4). Lethargy, progressing to somnolence and later coma, vomiting and refusal to feed, hypothermia, and seizures are common symptoms in neonates. Very often, the clinical situation is initially interpreted and treated as bacterial sepsis, which contributes to a significant delay in establishing the diagnosis if differential diagnoses are only considered after the failure of (in a day or two of) antibacterial treatment.

Outside the neonatal period, signs and symptoms become more variable but the neurological character remains. The reader is referred here to a comprehensive list of neonatal and late-onset signs and symptoms in the recently revised guideline paper (4).

The most relevant complication is brain damage, which can become irreversible already within a few hours after the occurrence of severe hyperammonaemia (21, 22). Although there is no clear threshold for plasma ammonia to cause irreversible brain damage, an elevation of ammonia exceeding 1,000 μmol/L for more than 24 h is in most cases associated with a poor prognosis and even early death of the patient (4). Making the clinical management of these patients very challenging, there are reports of single patients that survive such an episode without obvious neurological sequelae (23).

### Current Diagnostic Strategies

The most important diagnostic key is in the hands of those who first treat these patients. Doctors in the emergency departments or the neonatal intensive care units will be the first to manage a yet unknown neonatal patient with the first manifestation of hyperammonaemia. In the late-onset cases, doctors with different specialities, such as general physicians, neurologists, psychiatrists, gastroenterologists and also doctors in the emergency wards may see such patients first. These doctors are key to raise an early suspicion of an underlying metabolic defect and thereby recommend the analysis of plasma ammonia. It has been shown that neonates suffering from hyperammonaemia are much more likely to be tested for elevated blood lactate than for hyperammonaemia (24). This diagnostic algorithm must be improved giving the same importance to ammonia determination as to metabolites that can be tested applying point-of-care device such as lactate as a frequent part of standard blood gas analysis. Ideally, ammonia determination should be available to all units that are involved in the emergency management of patients of all ages.

If ammonia is found to be elevated, contact should be made to the next metabolic centre to discuss and plan the next diagnostic steps and initial therapy, and, depending on the clinical situation, the transfer of the patient for further management (4). In any patient with severe hyperammonaemia and indicative of significant neurological impairment with seizures or (pre) coma, an immediate transfer to a metabolic centre for further management including (the likely necessary) extrarenal ammonia detoxification using haemodialysis or haemodiafiltration should be considered and planned without delay.

If hyperammonaemia is found, then relevant laboratory analyses to understand its aetiology include the profiles of amino acids in plasma, organic acids in urine including the determination of orotic acid and acylcarnitines in the blood (25). With these ‘metabolic screening tests’, the majority of patients with symptomatic hyperammonaemia can be defined as a specific disorder, yet of course pending further confirmatory testing. For the group of primary hyperammonaemias, which is the focus of this manuscript, urinary organic acids (apart from increased urinary orotic acid in OTC deficiency and the distal UCDs) and blood acylcarnitines are mainly normal, while plasma amino acids frequently show specific changes. A summary of characteristic biochemical findings is listed in Table 1.

**Table 1:** Summary of the main biochemical aspects of primary hyperammonaemias

Primary hyperammonaemia			
Enzyme defects of the urea cycle			
Disorder	Main diagnostics	Key metabolites	Pathomechanism
NAGS deficiency	Amino acids in plasma	Glutamine in plasma increased, orotic acid in urine normal	Enzymatic block
CPS1 deficiency	Amino acids in plasma	Glutamine in plasma increased, orotic acid in urine normal	Enzymatic block
OTC deficiency	Amino acids in plasma, orotic acid in urine	Glutamine in plasma increased, orotic acid in urine increased	Enzymatic block
ASS deficiency	Amino acids in plasma, orotic acid in urine	Glutamine and citrulline in plasma increased, orotic acid in urine increased	Enzymatic block
ASL deficiency	Amino acids in plasma, orotic acid in urine	Glutamine, citrulline and argininosuccinate in plasma and urine increased, orotic acid in urine increased	Enzymatic block
ARG1 deficiency	Amino acids in plasma	Arginine in plasma increased	Enzymatic block

**Transporter defects of the urea cycle**			

HHH syndrome	Amino acids in plasma and urine	Ornithine in plasma increased, homocitrulline in urine increased	Deficiency of mitochondrial ornithine (substrate of OTC)
Citrin deficiency	Amino acids in plasma	Citrulline in plasma increased	Deficiency of cytosolic aspartate (substrate of ASS)

ARG1, arginase 1; ASL, argininosuccinate lyase; ASS, argininosuccinate synthetase; CPS1, carbamoylphosphate synthetase 1; HHH, hyperornithinemia-hyperammonaemia-hypercitrullinuria; NAGS, N-acetylglutamate synthase; OTC, ornithine transcarbamylase.

For confirmation of the diagnosis, mutation analysis is the preferred method, as it offers the additional advantage of specific testing of other family members (especially important in OTC deficiency) and prenatal testing in future pregnancies (4, 26). In addition to mutation analysis, functional tests can be done, mainly enzymatic analyses, but these require invasive samplings such as skin or liver biopsies and still lack the aforementioned additional advantages. Nevertheless, functional assays can be of high value in patients with inconclusive molecular genetic analysis (27).

Another diagnostic tool that was introduced more than 20 years ago is the use of stable isotopes for a flux study of the entire urea cycle (28, 29). This method was used in the past almost exclusively as part of the experimental studies involving novel therapy developments such as hepatocyte transplantation or gene therapy approaches (30), and these novel methods may facilitate future use of this approach (31). Establishing the ureagenesis capacity may also be useful in testing the relevance of the existing medications in new indications, for instance, the use of carglumic acid in UCDs other than NAGS deficiency. It has been proposed several times that stimulation of the urea cycle may also be beneficial for CPS1 deficiency, for example, but there is only preliminary experimental or clinical proof for such claims (32). The comparison of the urea cycle flux before and after the introduction of carglumic acid could help in identifying patients that benefit from this additional therapy.

### Current Therapeutic Strategies

Therapy of primary hyperammonaemia is discussed below separately for acute and chronic management.

*Acute management* is based on two principles: the rapid detoxification of ammonia and the establishment of anabolism to prevent any further protein breakdown (33). Detoxification of ammonia should be achieved as fast as possible to prevent (further) brain damage. The most powerful method for this is the extracorporeal removal of ammonia from the blood and organism by haemodialysis or haemodiafiltration (34, 35). The choice of the method will mainly depend on the local availabilities and experience. Drawbacks of any dialysis method are the time needed to start this procedure, potential adverse events associated with the insertion of vascular catheters, the necessary anticoagulation and disturbances of electrolytes and blood gas homoeostasis during the procedure. Therefore, any attempt should be made to identify patients early enough during metabolic decompensation(s) to avoid (or at least reduce) the need for such drastic therapies. However, patients identified with clinical signs of encephalopathy should be kept prepared for dialysis without any further delay. It has been shown that the majority of newborns with a presentation of any UCD will have to be subjected to dialysis (23). It is therefore not justified to first try a ‘conservative’ approach using only high glucose infusions ± insulin, nitrogen scavengers, and infusions of L-arginine to treat symptomatic hyperammonaemia.

*Chronic management* of primary hyperammonaemias is for most patients based on a combination of low protein diet plus the supplementation of essential amino acids, vitamins and trace elements to prevent their respective deficiencies, together with nitrogen scavengers and few other medications (4, 36). The list of medications, their usual dosages and the rationale are summarised in Table 2.

**Table 2:** Medications used in primary hyperammonaemias

Medications for acute and long-term use in primary hyperammonaemias			
Substance	Acute situation	Long-term use	Rationale
Sodium benzoate (infusion in 10% dextrose)	250 mg/kg bw as bolus in 90–120 min, then continuous infusion 250–500 mg/kg/day	250–500 mg/kg/day	Conjugation with glycine and excretion as non-toxic hippuric acid in urine
Sodium phenylbutyrate/ Sodium phenylacetate (infusion in 10% dextrose)	250 mg/kg bw as bolus in 90–120 min, then continuous infusion 250–500 mg/ kg/day	250–500 mg/kg/day	Conjugation with glutamine and excretion as non-toxic phenylacetylglutamine in urine
Glycerol phenylbutyrate	Not indicated	8.5 ml/m^2^/day (9.4 g/m^2^/day) in patients with body surface <1.3 m^2^ 7 ml/m^2^/day (8 g/m^2^/day) in patients with body surface ≥1.3 m^2^	Conjugation with glutamine and excretion as non-toxic phenylacetylglutamine in urine; slower release and uptake than sodium PBA
L-arginine hydrochloride^*^ (infusion in 10% dextrose)	250 mg/kg bw (1.2 mmol/kg) as bolus in 90–120 min, then continuous infusion 250 mg/kg/day	250 mg/kg/day	To improve the residual function of the urea cycle and avoid arginine deficiency
N-carbamyl-glutamate^#^ (tablet for oral/enteral use)	100 mg/kg as oral bolus (or via NG tube), then 25–62.5 mg/kg bw every 6 h	25–62.5 mg/kg bw every 6 h (taper to the lowest possible dose)	Activation of CPS1

bw, bodyweight; CPS1, carbamoylphosphate synthetase 1; NAGS deficiency, N-acetylglutamate synthase deficiency; NG, nasogastric; PBA, phenylbutyrate.

* Contraindicated in arginase deficiency.

# For NAGS deficiency.

## Outlook

As stated above, the outcomes of UCDs are currently far from ideal and novel therapies and are much needed. In past years, several novel therapeutic approaches have been proposed and some were even tested in preclinical models. However, only very few have made progress in clinical trials. In the following, some of those that were tested in clinical trials or are planned to be tested clinically are briefly discussed.

Hepatocyte or mesenchymal stem cell transplantations have been proposed as a ‘bridging’ therapy for severe UCDs, who are listed for liver transplantation (37, 38, 39). ‘Bridging’ intended to provide improved metabolic stability until liver transplantation. Initial human trials have indicated potential improvement, but they also have some drawbacks concerning their safety and eficacy. Currently, these trials are still ongoing (ClinicalTrials.govIdentifier: NCT03884959). As an alternative, adult human liver stem cells are currently evaluated in the clinic as a novel bridging technology applying a different source of liver cells (40).

Gene addition by using adeno-associated viral (AAV) vectors is currently back in clinical trials (ClinicalTrials.gov Identifier: NCT02991144) after some years of re-development of this approach following the tragic death of an adult OTC deficient patient who received an adenoviral vector causing the overwhelming immune reaction (41). AAV vectors have the advantage of being mainly hepatotropic, but in case of loss of eficacy in a dividing and growing liver, repeated administration of the same construct would not be possible for safety reasons (42). This may limit the use of this approach at least for neonatal, infant or small paediatric patients.

For the rare condition of arginase 1 (ARG1) deficiency, enzyme replacement with a pegylated form of arginase is currently evaluated in a clinical trial (ClinicalTrials.govIdentifier: NCT03921541). The underlying concept is based on the idea of an extensive arginine breakdown within the blood compartment allowing for the reduction of this amino acid in the entire organism, thus resulting in reduced neurological and hepatic toxicity (43). The long-term benefit of this approach and whether the neurological complications of this disease can be (partly) reverted or even prevented will likely determine the usefulness of this novel approach.

A diferent approach addressing the intestinal microbiome is currently part of phase 2, open-label trial in subjects with UCDs (ClinicalTrials.gov Identifier: NCT03933410). In this study, synthetic glycans are given to modify the bacterial colonic flora to reduce the urease-derived ammonia-reuptake in the large intestine.

It would be desirable that at least some of these trials turn out successful to reduce the burden of the disease still present in UCDs. Until then, with increased awareness, we all can contribute in taking care of patients with initial symptoms of hyperammonaemia.
